# Determinants of public environmental satisfaction: an analysis based on socio-ecological system theory

**DOI:** 10.3389/fpsyg.2025.1631240

**Published:** 2025-07-09

**Authors:** Bin Tang, Yuyao Tang, Fang Zhou

**Affiliations:** School of Public Administration, Xiangtan University, Xiangtan, China

**Keywords:** public environmental satisfaction, individual perception, institution and policy, social interaction, socio-ecological systems theory

## Abstract

**Introduction:**

Public environmental satisfaction, reflecting individuals’ evaluations of environmental conditions, is a key indicator of government performance in environmental governance.

**Methods:**

This study explores the determinants of public environmental satisfaction through the lens of socio-ecological systems theory, focusing on three analytical dimensions: the microsystem, mesosystem, and macrosystem. Drawing on data from the 2021 Chinese Social Survey, the analysis yields several important findings.

**Results:**

First, microsystem factors play the most significant role. Perceived environmental pollution notably reduces satisfaction, while satisfaction with government performance, personal life, and broader societal conditions significantly enhances it. Second, mesosystem factors have a comparatively weaker influence. Internet usage shows no significant effect, and passive or non-institutional forms of public participation are associated with lower satisfaction. Third, macrosystem factors—particularly institutional integrity, transparency of environmental information, and government responsiveness—positively and significantly shape public environmental satisfaction.

**Discussion:**

Overall, this study offers a comprehensive assessment of the multi-level influences on public environmental satisfaction and underscores the dominant role of individual-level perceptions. The findings provide valuable insights for policymakers seeking to strengthen environmental governance and improve public well-being.

## 1 Introduction

Environmental issues represent some of the most pressing threats to human health. According to the *World Health Statistics 2024*, millions of deaths each year are linked to environmental factors. Beyond their direct health implications, environmental problems have increasingly fueled social tensions, as evidenced by the global rise in protests against environmental degradation. At the core of these movements lies a deep dissatisfaction with the adverse effects of environmental conditions on public wellbeing.

Existing research has primarily examined the determinants of public environmental satisfaction from two perspectives: individual and contextual. On the individual level, scholars have explored how characteristics such as age (Ernst, 2019), gender (Su et al., 2018), educational attainment (Zhan et al., 2018), environmental awareness (Fu et al., 2020), and internet usage (Tang et al., 2021) influence perceptions of environmental quality and governance performance. On the contextual level, studies have emphasized the role of broader social and institutional factors, drawing on theoretical frameworks such as citizen participation (Glucker et al., 2013), social networks, social capital, and trust in government (Maor and Sulitzeanu-Kenan, 2016). While these studies highlight the significance of both individual and contextual influences, relatively few have examined how these multi-level factors interact to shape public environmental satisfaction. This gap limits our understanding of the heterogeneity in public perceptions and the diverse pathways through which satisfaction is formed. To address this shortcoming, the present study adopts a social-ecological systems framework to examine the combined effects of individual, social, and institutional factors on public environmental satisfaction. This approach not only enriches our understanding of public environmental concerns but also helps identify effective intervention points for improving environmental governance Ultimately, it contributes to advancing the national strategy of ecological civilization.

## 2 Theoretical framework and research hypotheses

### 2.1 Socio-ecological systems theory

Socio-ecological systems theory serves as a foundational framework for examining the interactions between individuals and their environments. This theory conceptualizes the social environment as a multi-tiered ecological system, highlighting the significance of social contexts in shaping human behavior. It categorizes the socio-ecological system into three distinct levels: the microsystem, mesosystem, and macrosystem. The microsystem pertains to individual members within the ecological system, focusing on personal characteristics and immediate environments. The mesosystem encompasses small-scale groups closely connected to individuals, such as families, peer groups, or occupational communities. In contrast, the “macrosystem includes broader societal structures, including cultures, communities, institutions, and organizations.” These three levels interact dynamically, collectively influencing individual behavior and perceptions.

The complexity, interrelatedness and spillover nature of ecological and environmental issues determine that the environmental conditions are naturally influenced by multiple factors, which also leads to the evaluation and attitude of individuals toward the environmental conditions presenting a complex and diverse nature. The public operates both as discrete individuals within the macrosystem and as collective entities engaging with organizations and societal structures. Consequently, public environmental satisfaction emerges from factors spanning all three ecological systems. Existing studies indicate that public environmental satisfaction is influenced by a diverse array of factors, including individual characteristics (Tang et al., 2023), environmental conditions, and institutional elements (Ahlers and Shen, 2018). Therefore, the social-ecological systems theory offers a suitable theoretical framework for analyzing the complex mechanisms underlying the formation of public environmental satisfaction.

Satisfaction fundamentally represents an individual’s assessment of the congruence between their lived experiences and established standards. Public environmental satisfaction refers to the subjective perception and recognition of the natural environment quality, pollution control effectiveness and ecological service functions of the area where the public lives. It is a core indicator for quantitatively assessing the match between the government’s environmental protection work and public demands. Environmental satisfaction can be divided into three domains: satisfaction with the surrounding environment, satisfaction with governmental environmental policies, and satisfaction with policies addressing specific environmental issues (Pelletier et al., 1996). While environmental satisfaction is inherently a personal evaluation, the microsystem level elucidates how biological, psychological, and social factors continuously interact throughout an individual’s life, shaping subjective perceptions and behaviors.

**FIGURE 1:**
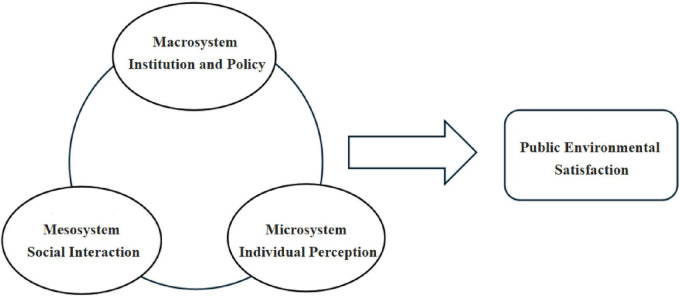
Research framework.

In practice, comprehending environmental satisfaction necessitates an analysis of the interplay among all ecological systems rather than isolating individual factors. For instance, dissatisfaction with environmental conditions may stem not only from personal perceptions of pollution but also from community-wide grievances or dissatisfaction with government performance. Acknowledging this complexity, the present study adopts an analytical framework that synthesizes the microsystem, mesosystem, and macrosystem dimensions (see Figure 1).

### 2.2 Research hypotheses

#### 2.2.1 Microsystem—individual perception

The microsystem emphasizes the role of individual perception in shaping attitudes and behaviors. It serves as the psychological foundation for environmental behavior: accurate perceptions of environmental conditions foster more rational responses. Individuals’ interpretations of local environmental changes directly influence their satisfaction with environmental quality (Davenport et al., 2019; Fernando et al., 2019). Dissatisfaction with environmental conditions or related government policies can negatively affect one’s environmental outlook, highlighting the importance of personal perception (Pelletier et al., 1996).

Previous research has identified several individual-level perceptions that significantly affect environmental satisfaction. These include subjective socioeconomic status (Tan et al., 2020; Kraus et al., 2017; Pickett and Wilkinson, 2015), interpersonal trust (Perry and Mankin, 2007; Guinot et al., 2014), trust in government (Chanley et al., 2000), perception of environmental pollution (Ju et al., 2021; Zhang et al., 2020; Kim and Park, 2013), and environmental self-efficacy (Hamann and Reese, 2020). These factors shape how individuals understand objective environmental conditions and ultimately influence their satisfaction levels. Accordingly, this study identifies six key microsystem variables: subjective socioeconomic status, interpersonal trust, trust in government, perceived environmental pollution, environmental self-efficacy, and overall satisfaction.

##### 2.2.1.1 Subjective socioeconomic status

Subjective socioeconomic status (SES) refers to an individual’s perceived position within the social hierarchy (Autin et al., 2017; Kraus et al., 2009). Individuals’ beliefs about their social standing influence how they access and interpret information, shaping their preferences and decisions. Those with higher subjective SES are generally more advantaged in social comparisons and report stronger feelings of relative gain. In this sense, higher perceived status is associated with more positive attitudes and greater satisfaction, including with environmental conditions.

##### 2.2.1.2 Interpersonal trust

Interpersonal trust reflects the value judgments individuals form through social interactions and denotes confidence in others’ behavior and intentions (De Jong and Elfring, 2010). High levels of trust generate both emotional and instrumental benefits, contributing to overall life satisfaction (Wang et al., 2019). Proactive individuals often cultivate stronger social relationships, which in turn enhance trust and satisfaction. Studies have shown that interpersonal trust is positively associated with life satisfaction (Bjørnskov, 2003; Dolan et al., 2008), prosocial behavior (Cadenhead and Richman, 1996; Yang et al., 2024), and job satisfaction (Matzler and Renzl, 2006; Perry and Mankin, 2007). In societies with high interpersonal trust, fostering trust can positively influence environmental satisfaction.

##### 2.2.1.3 Trust in government

Trust in government refers to public confidence in governmental institutions and their actions (Mishler and Rose, 1997). Empirical research consistently demonstrates a positive association between trust in government and environmental satisfaction. Higher trust levels lead to more favorable assessments of environmental governance efforts (Venkatesh et al., 2016) and enhance public support for environmental initiatives. Trust also influences satisfaction indirectly through perceived government performance and effectiveness (Ruan et al., 2022; Tang et al., 2023). Therefore, trust in government is a key determinant of public environmental satisfaction.

##### 2.2.1.4 Perception of environmental pollution

Perceived environmental pollution involves individuals’ subjective evaluations of environmental conditions, such as air and water quality. These perceptions bridge objective environmental realities and personal interpretations. Only when environmental degradation is perceived as a personal issue does it meaningfully affect satisfaction. Perceived exposure to pollution can elevate environmental concern, highlight health threats, and reduce satisfaction with environmental quality (Han, 2020). In fact, perception may have a stronger influence on environmental attitudes than broader social or cultural factors (Noel et al., 2021). Thus, perceived pollution is closely tied to environmental satisfaction.

##### 2.2.1.5 Overall satisfaction

This study defines overall satisfaction as a composite measure of satisfaction with life, government, and society. Life satisfaction reflects an individual’s long-term evaluation of their quality of life (De Vos, 2019). Government satisfaction assesses the gap between expectations and actual governmental performance (Oliver, 1980); when government performance meets or exceeds expectations, satisfaction increases. Social satisfaction refers to individuals’ evaluations of the broader societal environment. According to Putnam’s theory of social capital (Putnam, 1993; Putnam, 2000), trust, norms, and networks foster cooperative behavior and institutional efficiency. High social satisfaction promotes civic engagement in environmental governance, strengthens policy implementation, and enhances environmental satisfaction.

##### 2.2.1.6 Environmental self-efficacy

Environmental self-efficacy refers to individuals’ belief in their capacity to contribute to environmental improvement through personal action. It encompasses both perceived internal competence and subjective judgments about one’s ability to make a difference. Self-efficacy is a key motivator of environmental engagement. Moreover, it is influenced by perceptions of government responsiveness—i.e., the extent to which public concerns are addressed promptly and effectively. When individuals perceive government inaction or inefficiency, dissatisfaction grows. A stronger sense of self-efficacy enhances personal responsibility and is likely to increase environmental satisfaction.

In summary, this study investigates how microsystem-level factors—namely, subjective socioeconomic status, interpersonal trust, trust in government, perceived environmental pollution, overall satisfaction, and environmental self-efficacy—influence public environmental satisfaction. Based on this framework, the following hypotheses are proposed:

Hypothesis 1a: Subjective socioeconomic status is significantly and positively correlated with public environmental satisfaction. Individuals with higher subjective socioeconomic status exhibit greater environmental satisfaction.

Hypothesis 1b: Interpersonal trust is significantly and positively correlated with public environmental satisfaction. Higher levels of interpersonal trust lead to greater environmental satisfaction.

Hypothesis 1c: Trust in government is significantly and positively correlated with public environmental satisfaction. Greater public trust in government corresponds to higher environmental satisfaction.

Hypothesis 1d: Perception of environmental pollution is significantly and negatively correlated with public environmental satisfaction. Individuals who perceive more severe environmental degradation report lower environmental satisfaction.

Hypothesis 1e: Overall satisfaction is significantly and positively correlated with public environmental satisfaction. Individuals with higher life, government, and social satisfaction report greater environmental satisfaction.

Hypothesis 1f: Environmental self-efficacy is significantly and positively correlated with public environmental satisfaction. Public satisfaction increases as individuals’ perceived ability to contribute to environmental governance grows.

#### 2.2.2 Meso-system—social interaction

Social interaction fundamentally shapes individual preferences, expectations, and constraints through processes of mutual influence and interdependence (Durlauf and Ioannides, 2010). As Granovetter (2015) emphasizes, human behavior is often embedded in social networks, where information exchange and prevailing norms significantly guide individual actions. In the context of environmental and ecological issues, social interactions are essential for raising awareness, fostering discourse, and disseminating environmental information. For individuals lacking strong initial preferences, others’ opinions often serve as important cues that shape environmental perceptions and value orientations.

In digital spaces, the internet provides a key platform for accessing and exchanging information, enabling the public to stay informed about environmental policies and government performance. Consequently, internet usage may influence how individuals evaluate environmental governance and conditions. In physical social settings, reference groups and interpersonal interactions help create a social atmosphere that affects individuals’ emotions, values, and satisfaction. When people perceive that societal conditions fall short of their environmental expectations, they may resort to more intense or even risky forms of political participation. Based on these dynamics, this study posits that social interaction plays a significant role in shaping public environmental satisfaction.

##### 2.2.2.1 Internet usage

The relationship between internet use and public environmental satisfaction has attracted growing academic attention, though conclusions remain mixed. Two main perspectives dominate the literature. One view holds that internet use enhances public awareness and civic engagement (Zhao, 2012). By improving access to information and strengthening political support, internet use fosters more favorable evaluations of environmental governance.

In contrast, a second perspective suggests that increased internet use is linked to heightened concern over environmental issues and more negative assessments of environmental satisfaction (Yang et al., 2021). According to this view, digital media may distort public perceptions through selective reporting and sensationalism. In China, where environmental challenges are widespread, some media outlets exaggerate environmental incidents to attract attention and increase traffic. This amplifies the spread of negative environmental information, reinforcing pessimistic public perceptions. As a result, internet usage may lower satisfaction with both environmental conditions and governance. Based on this reasoning, this study hypothesizes that internet usage negatively influences public environmental satisfaction.

##### 2.2.2.2 Public participation

There is widespread agreement that public participation is essential to effective environmental governance. The International Association for Impact Assessment defines public participation as the involvement of individuals or groups affected by, or interested in, proposed policies or projects during the decision-making process. Participation is thus a core element of governance and significantly shapes public evaluations.

Procedurally, participation can be divided into “institutionalized” and “non-institutionalized” forms. Institutionalized participation refers to formal mechanisms such as voting and elections, through which citizens can express their preferences and influence policy. When such channels are accessible and functional, individuals are more likely to engage through these means. Non-institutionalized participation encompasses informal or *ad hoc* actions, such as submitting complaints to government agencies or expressing concerns through alternative, often grassroots, channels (Hooghe and Marien, 2013). This form typically arises when institutional options are perceived as ineffective, and may involve more confrontational or radical actions.

Importantly, it is not participation itself that affects public satisfaction, but rather the “perceived effectiveness” of the participation process. This includes whether individuals believe participatory channels are open, procedures are fair, and their voices are genuinely heard (Kweit and Kweit, 2007). Effective participation enhances public satisfaction by fostering a sense of inclusion and legitimacy. Conversely, symbolic or purely formal participation—where public input is neither welcomed nor considered—undermines trust and may prompt non-institutional expressions of dissatisfaction (Johnson, 2013; Sun, 2023). Such dynamics can erode satisfaction with both governance processes and environmental outcomes.

Building on this analysis, the study examines the influence of social interaction on public environmental satisfaction through two primary dimensions: “internet usage” and “public participation.” The following hypotheses are proposed:

Hypothesis 2a: During China’s current period of frequent environmental issues, internet usage is significantly and negatively correlated with public environmental satisfaction. Specifically, higher internet usage leads to lower environmental satisfaction.

Hypothesis 2b: There is a significant positive correlation between institutionalized participation and public environmental satisfaction, that is, the higher the degree of institutionalized participation, the higher the environmental satisfaction; while there is a significant negative correlation between non-institutionalized participation and public environmental satisfaction, that is, the higher the degree of non-institutionalized participation, the lower the environmental satisfaction.

#### 2.2.3 Macrosystem—institution and policy

At the macrosystem level, public environmental satisfaction is shaped by institutional structures and policy frameworks embedded in the broader social environment. Inadequate policy design or poor implementation often triggers public dissatisfaction with environmental conditions (Nie and Wang, 2023). While environmental pollution in China has not yet sparked large-scale unrest or nationwide movements, local communities are becoming increasingly aware of their rights and are demanding stronger protections for environmental and public health (Ahlers and Shen, 2018). This growing awareness has heightened public expectations for institutional performance and policy effectiveness. When government responses are perceived as inadequate, dissatisfaction increases, ultimately lowering environmental satisfaction.

Over the past 2 decades, environmental protection and pollution control have remained top priorities for China’s central government (Ahlers and Shen, 2018; Wang et al., 2021). Key reforms include the implementation of stricter environmental regulations, incorporation of environmental performance metrics into official evaluations, and efforts to enhance institutional legitimacy in environmental governance. These measures have led to notable improvements in pollution control (Karplus and Wu, 2019; Li et al., 2020). Within this context, the present study identifies three key macrosystem-level factors influencing public environmental satisfaction: institutional standardization, environmental information transparency, and government responsiveness.

##### 2.2.3.1 Institutional standardization

Well-established institutional norms and regulatory frameworks are essential for constraining the behavior of both governments and enterprises, thereby enhancing public perceptions of environmental governance (Lu and Wei, 2025). Research shows that in areas with stronger rule of law and oversight mechanisms, violations of environmental regulations are less frequent. For example, newly introduced ambient air quality standards have successfully improved corporate environmental performance—but only in provinces with effective enforcement (Zhang et al., 2022). These findings suggest that the degree of institutional standardization directly influences the strength and credibility of environmental governance, thereby shaping public satisfaction.

##### 2.2.3.2 Information transparency

Transparency in environmental information marks a shift away from traditional top-down management toward more participatory governance. It promotes public engagement, drives behavioral change, and addresses growing public expectations for openness. Effective public participation depends heavily on access to relevant and credible information (Czajkowski et al., 2016). Improved transparency enables citizens to voice preferences and make informed decisions, fostering higher levels of support for environmental initiatives (Chen and Cho, 2019).

Empirical studies support these claims. For instance, Tu et al. (2019), using a quasi-experimental design based on the Pollution Information Transparency Index, found that public disclosure of environmental data significantly contributes to local pollution reduction. Additionally, transparency enhances residents’ wellbeing, particularly when the disclosed information is perceived as credible (Zhu and Lin, 2022). Therefore, strengthening information transparency at the local level can bolster public trust and increase environmental satisfaction.

##### 2.2.3.3 Government responsiveness

Government responsiveness is a critical determinant of public satisfaction. Citizens’ evaluations of government performance are often based on the perceived quality of public services and the strength of government-citizen relationships (Glaser and Denhardt, 2000). When citizens feel meaningfully engaged in public affairs, positive interactions with government institutions build goodwill and enhance trust (Nie and Wang, 2023). In response to public concerns, governments may promote participation, increase accountability, and improve transparency. Such efforts have been shown to significantly enhance satisfaction with government performance (Ma, 2017). Furthermore, responsive and personalized interactions reduce perceived risks, foster psychological closeness, and strengthen institutional trust (Xue et al., 2020). Conversely, delays or inefficiencies in addressing public environmental demands can erode these positive effects. Empirical studies confirm that responsiveness significantly contributes to public environmental satisfaction (Nie and Wang, 2023).

Building on this analysis, the study investigates how macrosystem factors—including institutional standardization, information transparency, and government responsiveness—affect public environmental satisfaction. The following hypotheses are proposed:

Hypothesis 3a: Institutional standardization is positively correlated with public environmental satisfaction. The more standardized the government’s environmental governance efforts, the higher the level of public environmental satisfaction.

Hypothesis 3b: Information transparency is positively correlated with public environmental satisfaction. Greater transparency in government environmental governance leads to higher public environmental satisfaction.

Hypothesis 3c: Government responsiveness is positively correlated with public environmental satisfaction. The more responsive the government is to public environmental demands, the higher the level of public environmental satisfaction.

In summary, this study integrates the microsystem, mesosystem, and macrosystem dimensions to explore their effects on public environmental satisfaction. Institutional standardization, information transparency, and government responsiveness, as key components of the macrosystem, play a critical role in shaping public perceptions of environmental governance. By examining the interplay of these factors across multiple levels, the study aims to provide a comprehensive understanding of the determinants of public environmental satisfaction.

## 3 Data sources and research design

### 3.1 Data sources

This study relies on data from the 2021 China Social Survey (CSS2021), a nationally representative, continuous sampling survey initiated in 2005 by the Institute of Sociology, Chinese Academy of Social Sciences. The survey covers 31 provinces, autonomous regions, and municipalities across China, sampling 151 districts and counties as well as 604 villages or residential committees. After removing incomplete and invalid responses, the final dataset includes 2,518 valid samples. CSS employs a stratified multi-stage probability sampling method, constructing its sampling frame based on census data. However, population mobility may cause discrepancies between the sampling frame and actual population distribution, particularly manifesting in inadequate coverage of migrant populations and remote areas. Although CSS has enhanced sampling accuracy through a computer-assisted address sampling system, transient workers, populations without fixed residences, and other mobile groups may still be excluded, resulting in sample bias.

### 3.2 Variable selection and measurement

1. Dependent Variable: *Public Environmental Satisfaction*. Public environmental satisfaction plays a key role in promoting pro-environmental behavior. To facilitate scientific evaluation, Pelletier et al. (1996) developed the Environmental Satisfaction Scale, which includes two components: satisfaction with local environmental conditions and satisfaction with government environmental policies. This scale captures both the public’s evaluation of current environmental quality and their perception of government efforts in environmental governance. Following this framework, the present study measures public environmental satisfaction across two dimensions: satisfaction with environmental conditions and satisfaction with environmental governance. The final value is calculated as the average of the two, using a five-point Likert scale ranging from 1 (very dissatisfied) to 5 (very satisfied).

To measure *Satisfaction with Environmental Quality*, the study uses item *D5b* from the CSS2021, which asks “*How satisfied are you with the current environmental quality in your place of residence?*” The original responses were recoded into a five-point Likert scale, where higher values represent greater satisfaction.

To measure *Satisfaction with Environmental Governance*, the study uses item *G3-3: “How well is the government performing in protecting the environment and controlling pollution*?” Responses coded as “8” (i.e., “Hard to say”) were treated as neutral and assigned a score of 3, corresponding to “average.” These responses were also converted into a five-point Likert scale, with higher scores indicating higher satisfaction.

2. Independent Variables. The independent variables are categorized into three dimensions based on the socio-ecological systems theory: individual perceptions (microsystem level), social interaction (mesosystem level), and institutions and policies (macrosystem level). Each dimension includes multiple factors and corresponding survey items.

*Microsystem Level – Individual Perceptions*. This dimension examines how personal evaluations and experiences shape satisfaction. The key factors include: (1) *Subjective Socioeconomic Status*, measured by item “*D3a:* What is your perceived socioeconomic status in your local area?” (2) *Interpersonal Trust*, measured by item “*F1b:* Evaluation of the current level of trust among people.” (3) *Trust in Government*, measured by items F1a-1, F1a-2, and F1a-3, which assess trust in the central, district/county, and township governments, respectively. (4) *Perceived Environmental Pollution*, measured by item *D5a-1*, *D5a-1*, *D5a-2*, and *D5a-3*, which assess perceptions of air pollution, water quality, and noise pollution. (5) *Overall Satisfaction*, measured by items *D2a-6* (life satisfaction), *G3-14* (evaluation of local government performance), and *G6* (evaluation of society overall). (6) *Environmental Self-efficacy*, measured by item “*D5c-4*: I lack knowledge or capability to comment on environmental issues.”

*Mesosystem Level – Social Interaction*. This dimension focuses on how individuals engage with their social environment. The main factors include: (1) *Internet Usage*, measured by items *D4b1-1* through *D4b1-7*, which assess activities such as browsing current affairs, entertainment, socializing, work-related tasks, learning, online shopping, and financial management. Internet usage is reverse-coded so that higher scores indicate greater frequency of use. (2) *Political Participation*, measured by items *H1a-1*, *H1a-2*, and *H1a-5*, which assess activities such as sharing opinions via media, reporting issues to government departments, and participating in collective action.

*Macrosystem Level – Institutions and Policies*. This dimension evaluates the institutional and policy environment. Relevant factors include: (1) *Institutional Normativity*, measured by item *G3-7*, “Rule of law and fair enforcement of regulations.” (2) *Information Transparency*, measured by item *G3-10*, “Openness of information and transparency in government operations.” (3) *Government Responsiveness*, measured by item *G3-11*, “Service orientation and timely response to public needs.” Because the original survey items vary in terms of their directionality, reverse coding is applied to several variables to ensure consistency across dimensions. Specifically, subjective socioeconomic status, perceived environmental pollution, satisfaction with government, internet usage, and the three macrosystem indicators are reverse-coded and keep the coding consistent with other variables. Details of this coding process are provided in Table 1.

**TABLE 1:** CSS2021 variables and descriptive statistics.

Variable			Survey item and operationalization		Mean	SD
**Control variables**						
Gender			Item a1b1-a: male = 1, female = 2		1.53	0.50
Age			Item a1c1: 2021 minus year of birth		42.39	12.94
Education level			Item a1d1: educational attainment based on questionnaire scale		4.39	2.14
Political affiliation			Item A3: communist party member = 1, others = 2		1.86	0.35
Household registration type			Item A4a: agricultural registered permanent residence = 1, non-agricultural registered permanent residence = 2		1.39	0.49
Personal total income			Item B8a: log-transformed total personal income		10.06	1.32
Household total income			Item C4a: log-transformed total household income		10.99	1.14
**Independent variables Micro-system**						
Subjective socioeconomic status			Item D3a: subjective socioeconomic status (reverse-coded)		2.42	0.89
Interpersonal trust			Item F1b: evaluation of interpersonal trust level		6.30	1.92
Trust in government [converted to a 5-point scale]			Item F1a-1: trust in central government		4.48	0.78
			Item F1a-2: trust in district/county government		3.71	1.16
			Item F1a-3: trust in township government		3.46	1.25
Perceived environmental pollution [5-point scale conversion, reverse-coded]			Item D5a-1: air pollution		2.38	1.28
			Item D5a-2: water pollution		2.39	1.31
			Item D5a-3: noise pollution		2.23	1.29
Overall satisfaction			Item D2a-6: life satisfaction		7.22	2.01
			Item G3-14: government satisfaction (5-point scale conversion, reverse-coded)		3.78	0.94
			Item G6: social satisfaction		7.25	1.62
Environmental self-efficacy			Item D5c-4: I lack knowledge or capability to comment on environmental issues	[5-point scale conversion]	3.49	1.31
**Meso-system**						
Internet usage [reverse-coded]			Item D4b1-1: browsing current affairs		3.58	1.77
			Item D4b1-2: entertainment and leisure		3.61	1.73
			Item D4b1-3: chatting and socializing		3.90	1.57
			Item D4b1-4: business or work-related activities		2.16	2.24
			Item D4b1-5: educational and learning activities		2.16	2.04
			Item 4b1-6: online shopping		1.75	1.60
			Item 4b1-7: investment and financial management		0.33	0.99
Public participation		Institutionalized participation	H1a-3: participating in village (neighborhood) committee elections		1.32	0.47
			H1a-4: participating in major decision-making discussions of the village/unit where they reside.		1.11	0.32
		Non-institutionalized participation	Item H1a-1: reflecting opinions via newspapers/radio/online forums		1.04	0.20
			Item H1a-2: reporting issues to government departments		1.09	0.29
			Item H1a-5: participating in online/offline collective action		1.05	0.21
**Macro-system**						
Institutional normativity			Item G7: rule of law and fair enforcement of regulations	[5-point scale conversion, reverse-coded]	3.67	1.07
Information transparency			Item G10: openness of information and government transparency		3.50	1.13
Government responsiveness			Item G10: service orientation and timely response to public needs		3.43	1.17
**Dependent variable**						
Public environmental satisfaction			Average of satisfaction with environmental status and governance		3.74	1.10

3. To simplify the structure of the independent variables and reduce the risk of multicollinearity, exploratory factor analysis was conducted on the multi-item constructs of perceived environmental pollution, trust in government, overall satisfaction, internet usage, and political participation. For *perceived environmental pollution*, the Kaiser-Meyer-Olkin (KMO) value is 0.665, and Bartlett’s test of sphericity is significant (*p* < 0.001), indicating suitability for factor analysis. For *trust in government*, the KMO value is 0.762 and the Bartlett test is also significant (*p* < 0.001). For *overall satisfaction*, the KMO value is 0.609 with a significant Bartlett test (*p* < 0.001). For *internet usage*, the KMO value is 0.770 and Bartlett’s test is significant (*p* < 0.001). Two factors are extracted: the first is *information acquisition*, which includes learning and education, business and work, browsing current affairs, and online shopping or life services; the second is *entertainment activities*, which include chatting, socializing, and recreational use.

For *political participation*, the KMO value is 0.605 and Bartlett’s test is significant (*p* < 0.001). Two factors are extracted: the first is *non-institutionalized participation*, which includes reporting issues to newspapers, radio, or online platforms, contacting government departments, and participating in online or offline collective actions; the second is *institutionalized participation*, which includes voting in village/community elections and participating in major discussions within one’s residential community or workplace. Overall, based on the KMO values and the significance of Bartlett’s tests, factor analysis is appropriate for all selected constructs.

4. Control Variables. The study controls for key sociodemographic characteristics based on prior research. Control variables include *gender*, *age*, and *education level*. *Political affiliation* is coded as “Communist Party member = 1, others = 2.” Both *personal and household income* are log-transformed for analysis. Table 1 provides detailed descriptions, operationalizations, and descriptive statistics for these variables.

### 3.3 Analytical tools and models

To determine the factors influencing public environmental satisfaction, the study uses STATA 17.0 for model estimation. Since both the dependent and independent variables are continuous, and the purpose is to analyze the relationship between one continuous dependent variable and multiple continuous independent variables, the core assumption of which is that there is a linear relationship between the independent variable and the dependent variable, this paper adopts a multiple regression analysis model, because the dependent variable may be jointly affected by multiple independent variables, while the multiple regression controls the influence of other variables. The independent contribution of an independent variable to a dependent variable can be estimated more accurately and is easy to interpret. At the same time, robustness tests (such as residual analysis and multicollinearity test) can be used to ensure the reliability of the model. Therefore, this paper incorporated predictor variables into the model sequentially to explore the influence of different dimension factors. The regression model is specified as:


$$S\,a\,t\,i\,s\,f\,a\,c\,t\,i\,o\,n=\beta_{0}+\beta_{1}\,x_{1}+\beta_{2}\,x_{2}+\ldots +\beta_{i}\,x_{i}+\mu$$

where *x*_*i*_ represents the *i*-th control or independent variable, such as age, income, political affiliation, individual perceptions, government performance, or social interaction. β*_0_* is the intercept, β*_*i*_* represents the regression coefficient for the *i*-th variable, and μ denotes the random error term.

## 4 Analysis and findings

### 4.1 Multicollinearity testing

Prior to conducting the regression analysis, this study assessed potential multicollinearity among the independent variables by calculating the Variance Inflation Factor (VIF). All VIF values ranged between 1 and 10, the standard error of each variable was small, no abnormal expansion occurred, the risk of collinearity was low, and the *F*-value was significant, indicating that the model was effective as a whole, and the collinearity did not significantly weaken the explanatory power. It shows that there is no multicollinearity between the variables, and multiple linear regression can be used for analysis. This finding validates the appropriateness of employing multiple linear regression for the subsequent analysis.

To identify the determinants of public environmental satisfaction, the study initially employed a multiple linear regression model, incorporating variables from three dimensions: microsystem, mesosystem, and macrosystem. To further ensure the robustness of the results, an Ordered Logit model was also utilized for a supplementary robustness check. The outcomes of these analyses are detailed in Tables 2, 3.

**TABLE 2:** Multivariate regression analysis of multidimensional factors influencing public environmental satisfaction.

Variables			Public environmental satisfaction				
			M1	M2	M3	M4	(95% CI)
**Control variables**							
Gender			-0.025 (0.045)	-0.002 (0.038)	-0.024 (0.046)	-0.039 (0.040)**	0.921 (0.799, 1.061)
Education level			-0.055 (0.014)**	-0.071 (0.012)***	-0.065 (0.014)**	-0.035 (0.012)	0.975 (0.935, 1.017)
Household registration type			0.034 (0.053)	0.035 (0.044)*	0.047 (0.053)**	0.026 (0.047)	0.968 (0.820, 1.142)
Age			0.004 (0.002)	0.000 (0.002)	-0.004 (0.002)	0.017 (0.002)	0.997 (0.991, 1.003)
Political affiliation			-0.087 (0.069)***	-0.025 (0.058)	-0.067 (0.070)***	-0.051 (0.062)***	0.659 (0.531, 0.819)***
Personal total income			-0.015 (0.019)	-0.004 (0.016)	-0.018 (0.019)	-0.018 (0.017)	0.971 (0.915, 1.031)
Household total income			0.010 (0.022)	-0.006 (0.018)	0.010 (0.022)	0.015 (0.020)	1.001 (0.937, 1.069)
**Independent variables**							
**Micro-system**							
Subjective socioeconomic status				0.036 (0.022)**			1.092 (1.002, 1.19)*
Interpersonal trust				0.064 (0.011)***			1.161 (1.110, 1.215)***
Trust in government				0.105 (0.022)***			1.053 (0.969, 1.144)
Perceived pollution				-0.360 (0.015)***			0.383 (0.353, 0.416)***
Environmental self-efficacy				0.003 (0.015)			1.078 (1.017, 1.143)*
Overall satisfaction				0.260 (0.023)***			1.846 (1.677, 2.032)***
**Meso-system**							
Internet usage		Information acquisition			0.038 (0.050)		1.107 (1.010, 1.213)*
		Entertainment activities			0.004 (0.058)		0.950 (0.876, 1.030)
Public participation		Institutionalized participation			0.090 (0.048)***		0.977 (0.912, 1.047)
		Non-institutionalized participation			-0.051 (0.043)**		1.483 (1.229, 1.790)***
**Macro-system**							
Institutional normativity						0.195 (0.023)***	1.236 (1.138, 1.342)***
Information transparency						0.161 (0.024)***	1.155 (1.060, 1.260)**
Government responsiveness						0.163 (0.024)***	1.210 (1.111, 1.317)***
*R* ^2^			0.010	0.321	0.021	0.209	
Adjust *R*^2^			0.007	0.317	0.017	0.205	
*F*			3.685**	91.227***	4.944***	60.260***	

Standardized regression coefficients are presented before the estimates, with standard errors displayed in parentheses. Significance levels are indicated as follows: ***p < 0.01, **p < 0.05, and *p < 0.1.

**TABLE 3:** Ordered logistic regression analysis of multidimensional factors affecting public environmental satisfaction.

Variables		Public environmental satisfaction			
		M5	M6	M7	M8
**Control variables**					
Gender		-0.093 (0.090)	-0.008 (0.099)	-0.010 (0.092)	-0.127 (0.096)
Education Level		-0.046 (0.027)*	-0.087 (0.030)***	-0.051 (0.029)*	-0.052 (0.029)*
Household registration type		0.262 (0.107)**	0.316 (0.116)***	0.313 (0.108)***	0.251 (0.114)**
Age		0.002 (0.004)	0.006 (0.005)	0.001 (0.005)	0.004 (0.004)
Political affiliation		-0.444 (0.149)***	-0.202 (0.160)	-0.350 (0.152)**	-0.282 (0.158)*
Personal total income		0.007 (0.037)	0.029 (0.040)	0.002 (0.038)	0.007 (0.039)
Household total income		0.007 (0.044)	-0.030 (0.049)	0.011 (0.044)	0.005 (0.047)
**Independent variables**					
**Micro-system**					
Subjective socioeconomic status			0.143 (0.079)*	
Interpersonal trust			0.244 (0.076)***	
Trust in government			0.420 (0.080)***	
Perceived environmental pollution			-0.837 (0.053)***	
Environmental self-efficacy			-0.006 (0.055)	
Overall satisfaction			0.532 (0.068)***	
**Meso-system**					
Internet usage	Information acquisition		0.112 (0.101)		
	Entertainment activities		0.011 (0.118)		
Public participation	Institutionalized participation		0.323 (0.101)***		
	Non-institutionalized participation		-0.267 (0.081)***		
**Macro-system**					
Institutional normativity					0.417 (0.059)***
Information transparency					0.383 (0.063)***
Government responsiveness					0.368 (0.062)***
Pseudo *r*-squared		0.006	0.129	0.012	0.101

Standardized regression coefficients are presented before the estimates, with standard errors displayed in parentheses. Significance levels are indicated as follows: ***p < 0.01, **p < 0.05, and *p < 0.1.

### 4.2 Regression results analysis

The regression analysis includes four models. Model M1 contains only control variables, while Models M2, M3, and M4 sequentially introduce variables from the microsystem, mesosystem, and macrosystem levels to assess their respective impacts on public environmental satisfaction.

The results from Model M1 show that among the control variables, only political affiliation and education level are significantly associated with public environmental satisfaction, both displaying negative effects. Specifically, members of the Communist Party report higher levels of environmental satisfaction than non-members. This may reflect the advantages often enjoyed by Party members, such as higher socioeconomic and political status, which contribute to better living and working environments. Education level also has a negative effect on environmental satisfaction. A possible explanation is that individuals with higher education tend to develop stronger critical thinking and social responsibility, which makes them more sensitive to environmental risks and more demanding of environmental governance. These individuals, often referred to as “critical citizens,” are likely to hold higher expectations for environmental quality, resulting in more critical evaluations and lower satisfaction (Tang et al., 2021).

Model M2 evaluates microsystem-level variables and identifies several key determinants of public environmental satisfaction. First, *perceived environmental pollution* significantly reduces satisfaction, with the relationship passing the 1% significance level. This result supports *Hypothesis 1d*, suggesting that individuals who perceive more severe air, water, or noise pollution are less satisfied with the environment. For every unit increase in environmental pollution perception, public environmental satisfaction decreased by 0.36 times. Second, *overall satisfaction*, which reflects evaluations of life, society, and government, has a strong positive effect on *environmental satisfaction*, also significant at the 1% level. For every unit increase in overall satisfaction, public environmental satisfaction increases 0.26 times. This finding confirms *Hypothesis 1e*, indicating that those who are generally more satisfied with their broader circumstances tend to view environmental conditions more favorably. Additional microsystem factors, including *subjective socioeconomic status*, *interpersonal trust*, and *trust in government*, positively influence *public environmental satisfaction*. These effects are statistically significant at the 5, 1, and 1% levels, respectively, and for each unit increase of the three variables, public environmental satisfaction increases 0.036 times, 0.064 times and 0.105 times. providing support for Hypotheses *1a, 1b*, and *1c*. However, *environmental self-efficacy*, while showing a positive relationship, is not statistically significant, and thus *Hypothesis 1f* is not supported. One plausible explanation is that limited public awareness of environmental issues weakens individuals’ ability to assess their own role in environmental governance. This highlights the complex nature of self-efficacy as a psychological construct involving attitudes, emotions, and beliefs.

Model M3 evaluates mesosystem-level factors. The results show that institutionalized participation is positively associated with environmental satisfaction and is significant at the 1% level. In contrast, non-institutionalized participation is negatively associated with satisfaction and is significant at the 5% level. These findings support Hypothesis 2b but contrast with some studies that suggest public participation generally enhances satisfaction. One possible explanation is that institutionalized participation—such as voting, public hearings, and community involvement—provides channels for individuals to express their views and feel respected, which increases satisfaction. By contrast, public awareness of participation rights in China remains limited. Citizens often avoid engaging unless problems become severe, at which point they may resort to more reactive or confrontational forms of participation. Many environmental protests and mass incidents are examples of such non-institutionalized engagement triggered by dissatisfaction.

Moreover, public participation in China continues to face structural constraints. Issues such as vague central policies, weak legal enforcement, limited government feedback, and the exclusion of public opinion from performance evaluations often undermine the effectiveness of participation processes. In many cases, participation becomes symbolic rather than substantive, leading to formalistic implementation and further dissatisfaction. Regarding internet usage, neither of the two extracted factors—information acquisition and entertainment—has a statistically significant effect on environmental satisfaction. As a result, Hypothesis 2a is not supported. This may be due to the uneven quality and credibility of environmental information available online, which can be diluted by misinformation, conflicting narratives, and information overload. Furthermore, individual differences in media preferences and interpretation may distort the influence of online environmental content. Additionally, the study’s measurement of internet usage was limited in scope, potentially introducing measurement bias and narrowing the concept.

Model M4 assesses macrosystem-level variables and highlights the significant positive effects of *institutional normativity*, *information transparency*, and *government responsiveness* on *public environmental satisfaction*. For each one-unit increase in institutional normativity, public environmental satisfaction rises by 0.195 units. Similarly, a one-unit improvement in information transparency increases satisfaction by 0.161 units, while a one-unit improvement in government responsiveness raises satisfaction by 0.163 units. All three factors pass the 1% significance test, confirming *Hypotheses 3a, 3b*, and *3c*. These results demonstrate that as institutions become more standardized, transparent, and responsive, public satisfaction with environmental quality and governance improves.

A comparison of the four models reveals the relative explanatory power of the three dimensions. Microsystem variables account for the largest share of variance in public environmental satisfaction, explaining 31.7%. This underscores the importance of individual perceptions in shaping satisfaction levels. In contrast, mesosystem variables contribute the least, explaining only 1.7% of the variation. In order to prevent the coefficient from being affected by extreme values, this paper checks whether there are extreme scores (such as all 0 or 10) in the original variable, but no abnormalities are found. In order to further verify the regression results, the robustness test is also carried out.

### 4.3 Robustness test

Given that the dependent variable—public environmental satisfaction—is an ordered categorical variable, the Ordered Logit model offers a more appropriate estimation framework. By modeling cumulative probabilities, it better reflects the underlying data structure. To ensure the reliability of the results, this study conducts a robustness check using an Ordered Logit model. This approach addresses potential model specification bias stemming from measurement error in the dependent variable and provides complementary insights from a non-linear analytical perspective. For the Ordered Logit estimation, the environmental satisfaction variable is recoded into three categories: dissatisfied, neutral, and satisfied. The parallel lines assumption, a key requirement of the model, was tested using the Oparallel test. All variables met the assumption, with *p*-values exceeding 0.05, indicating that the model specification is appropriate. The regression results are presented in Table 3.

The robustness test results are highly consistent with those from the multiple linear regression models. Model M5, which includes only control variables, shows that both political affiliation and education level have significant negative effects on public environmental satisfaction, significant at the 10 and 1% levels, respectively. These findings are in line with those of Model M1. In Model M6, microsystem-level variables are added. The results confirm that *subjective socioeconomic status, interpersonal trust, trust in government, perceived environmental pollution*, and *overall satisfaction* all exert statistically significant effects on *public environmental satisfaction*, significant at the 10, 1, 1, 1, and 1% levels, respectively. These findings are consistent with those from the linear regression analysis and reaffirm Hypotheses 1a through 1e.

Model M7 examines mesosystem-level effects. *Internet usage* continues to show a positive but statistically insignificant effect on environmental satisfaction, thus failing to support Hypothesis 2a. However, *institutionalized participation* remains significantly and positively associated with environmental satisfaction, while *non-institutionalized participation* shows a significant negative effect. Both results are significant at the 1% level, mirroring the findings of the linear regression model and further supporting Hypothesis 2b. Model M8 evaluates macrosystem variables and corroborates the findings from Model M4. *Institutional normativity*, *information transparency*, and *government responsiveness* all significantly and positively influence *public environmental satisfaction*, and all pass the significance test of 0.01, which is basically consistent with model 3 of multiple linear regression analysis. These results once again confirm *Hypotheses 3a, 3b*, and *3c*.

Overall, the Ordered Logit regression results demonstrate strong consistency with the multiple linear regression findings. This consistency underscores the robustness of the study’s conclusions and highlights the reliability of the identified relationships between public environmental satisfaction and the influencing factors across micro-, meso-, and macro-level dimensions.

## 5 Conclusion

Drawing on data from CSS2021, this study employs a multiple linear regression model within the framework of social ecosystem theory to analyze public environmental satisfaction across three dimensions: microsystem, mesosystem, and macrosystem. The findings are as follows. First, the microsystem emerges as the most influential dimension affecting public environmental satisfaction. Among microsystem variables, perceived environmental pollution exerts the strongest impact. Second, the mesosystem has a relatively weaker effect, with only one variable—social participation—showing a significant correlation with public environmental satisfaction, and that correlation is negative. Third, the macrosystem’s influence lies between that of the microsystem and mesosystem—stronger than the mesosystem but weaker than the microsystem.

## 6 Discussion

Public environmental satisfaction is influenced by a variety of factors. A comprehensive analytical framework is therefore essential, and the social ecosystem theory provides a valuable lens for such analysis. Grounded in China’s governance context, this study adopts the classification structure of social ecosystem theory to categorize influencing factors into macro-, meso-, and micro-level dimensions. The findings reveal that the microsystem exerts the strongest explanatory power, underscoring the central role of individual perception in shaping public environmental satisfaction. This aligns with existing literature and supports the hypotheses proposed in this study. In addition to the microsystem, the macrosystem also has a significant impact, highlighting the importance of environmental institutions and policies as key public concerns and benchmarks for assessing environmental conditions.

As environmental awareness increases, dissatisfaction is increasingly expressed through concrete actions rather than merely verbal complaints—an observation reflected in the study’s results. At the mesosystem level, different forms of participation produce contrasting effects: institutionalized participation is positively associated with environmental satisfaction, while non-institutionalized participation has a significant negative impact. As noted by previous research, social participation can be classified as either effective or ineffective, with only the former yielding positive outcomes. In essence, it is not participation itself that drives satisfaction, but rather the perceived effectiveness of the process. This perception depends on whether participation channels are accessible, procedures are fair, and individual voices are acknowledged (Kweit and Kweit, 2007).

In practice, public engagement in China’s environmental governance tends to be reactive, often triggered by acute dissatisfaction with environmental conditions. Citizens may resort to complaints, protests, or other non-institutionalized actions. Structural constraints—such as unclear political signals from the central government, limited enforceability of environmental laws, and weak incentives for local governments—frequently reduce public participation to a symbolic act. In many cases, governments fail to treat public input as a genuine tool for incorporating public opinion and collective wisdom. As a result, participation becomes formalized rather than substantive, undermining its effectiveness and potentially exacerbating public discontent (Ma and Yang, 2019). These dynamics may further diminish public environmental satisfaction. Overall, most of the study’s hypotheses are supported, with the exception of Hypothesis 1f, which was not statistically validated.

Based on these findings, the study offers several policy recommendations to enhance public environmental satisfaction. First, participation mechanisms should be improved through institutional innovation, diversification of engagement formats, and the establishment of effective feedback systems to ensure that public concerns are meaningfully addressed. Second, environmental information disclosure should be strengthened. Pollution data and governance updates should be published in a timely manner via a centralized platform to protect citizens’ right to information. Third, government responsiveness should be enhanced. A smart dispatch system could be developed to categorize and route public requests to the appropriate departments, track resolution progress, and ensure timely responses. Complex issues should be addressed through coordinated interdepartmental collaboration, with full transparency of both process and outcomes, and integrated feedback mechanisms for public evaluation.

By applying social ecosystem theory to systematically examine the multidimensional drivers of environmental satisfaction, this study contributes to a more holistic understanding of the issue and helps clarify ongoing debates about the relative impact of different factors. While previous studies have examined these variables individually, few have conducted cross-dimensional comparisons. This research finds that both microsystem and macrosystem factors significantly influence public environmental satisfaction, with the microsystem playing the most decisive role.

Several limitations of this study should be acknowledged. First, the analysis is based on China’s specific governance context, and its generalizability to other national contexts remains uncertain. Although the CSS team employed computer-assisted address sampling and weighting techniques to minimize error, sample coverage remains a challenge—particularly when it involves rural areas, mobile populations, and cross-group comparisons we should be alert the overgeneralization of conclusions. Second, the data were collected in 2021, and more recent data are needed to validate the relationships identified in this study. Third, environmental self-efficacy was measured using a single survey item, which may not fully capture the construct’s multidimensional and context-sensitive nature. Data limitations constrained the depth of measurement. Finally, due to the availability of indicators, this study was unable to systematically explore more complex interactions between additional variables and public environmental satisfaction. Future research should further investigate these relationships.

## Data Availability

The original contributions presented in the study are included in the article/supplementary material, further inquiries can be directed to the corresponding author.
